# Effect of antiretroviral therapy on malaria incidence in HIV-infected Ugandan adults

**DOI:** 10.1097/QAD.0000000000001344

**Published:** 2017-02-01

**Authors:** Ronnie P. Kasirye, Heiner Grosskurth, Paula Munderi, Jonathan Levin, Zacchaeus Anywaine, Andrew Nunn, Anatoli Kamali, Kathy Baisley

**Affiliations:** a MRC/UVRI Uganda Research Unit on AIDS, Entebbe, Uganda; b London School of Hygiene and Tropical Medicine, London, UK; c School of Public Health, University of Witwatersrand, Johannesburg, South Africa; d MRC Clinical Trials Unit at University College London, UK.

**Keywords:** antiretroviral therapy, cotrimoxazole, HIV, malaria

## Abstract

**Introduction:**

Using the data of a trial on cotrimoxazole (CTX) cessation, we investigated the effect of different antiretroviral therapy (ART) regimens on the incidence of clinical malaria.

**Methods:**

During the cotrimoxazole cessation trial (ISRCTN44723643), HIV-infected Ugandan adults with CD4^+^ at least 250 cells/μl were randomized to receive either CTX prophylaxis or placebo and were followed for a median of 2.5 years. Blood slides for malaria microscopy were examined at scheduled visits and at unscheduled visits when the participant felt unwell. CD4^+^ cell counts were done 6-monthly. Malaria was defined as fever with a positive blood slide. ART regimens were categorized as nucleoside reverse transcriptase inhibitor (NRTI) only, non-nucleoside reverse transcriptase inhibitor (NNRTI)-containing or protease inhibitor containing. Malaria incidence was calculated using random effects Poisson regression to account for clustering of events.

**Results:**

Malaria incidence in the three ART regimen groups was 9.9 (3.6-27.4), 9.3 (8.3-10.4), and 3.5 (1.6-7.6) per 100 person-years, respectively. Incidence on protease inhibitors was lower than that on the other regimens with the results just reaching significance (adjusted rate ratio 0.4, 95% confidence interval = 0.2–1.0, comparing with NNRTI regimens). Stratification by CTX/placebo use gave similar results, without evidence of an interaction between the effects of CTX/placebo use and ART regimen. There was no evidence of an interaction between ART regimen and CD4^+^ cell count.

**Conclusion:**

There was some evidence that protease inhibitor-containing ART regimens may be associated with a lower clinical malaria incidence compared with other regimens. This effect was not modified by CTX use or CD4^+^ cell count. The antimalarial properties of protease inhibitors may have clinical and public health importance.

## Introduction

Antiretroviral therapy (ART) is used to control HIV replication in infected patients [1,2]. In addition, some ART drugs, particularly protease inhibitors, have shown antimalaria properties *in vitro*[3–5]. In children, a randomized clinical trial showed that use of protease inhibitor ART was associated with a lower risk of recurrent malaria compared with non-nucleoside reverse transcriptase (NNRTI)-based ART [6]. However, a study of HIV infected adult women found no beneficial effect of lopinavir/ritonavir compared with nevirapine on malaria incidence [7]. Studies on protease inhibitor use in the general adult population are lacking.

The present study undertook a subanalysis of data collected in the COSTOP (cotrimoxazole cessation) trial (ISRCTN44723643), a study designed to investigate the safety of stopping cotrimoxazole (CTX) in HIV-infected adults stable on ART, to assess whether malaria incidence differed between participants receiving different ART regimens and whether any such effects were modified by CTX use or CD4^+^ cell count.

## Methods

The study was conducted among COSTOP trial participants in Uganda, a country with high malaria endemicity [8,9]. COSTOP has been described previously [10,11]. In brief, this randomized placebo controlled noninferiority trial was conducted between 2011 and 2014 to determine whether prophylaxis with CTX can be safely discontinued among HIV infected adults on ART with CD4^+^ cell counts at least 250 cells/μl.

Participants were followed every month for the first 3 months and 3-monthly, thereafter, for a median of 2.5 years. At enrolment participants were provided with an insecticide-treated bed net. Blood samples for malaria microscopy were collected at scheduled visits and unscheduled visits if malaria was suspected. CD4^+^ cell counts were done 6-monthly. Participants diagnosed with malaria were treated in accordance with the national guidelines [12]. Participants were encouraged to return to the study clinics if they felt unwell, those treated elsewhere were asked to present documentary evidence of diagnoses: if no documentation was available this was not considered a confirmed case. Malaria for this analysis was defined as a history of fever with parasitaemia on blood slide. Presumptive malaria cases (blood slide negative or not done) were not included.

**Fig. 1:**
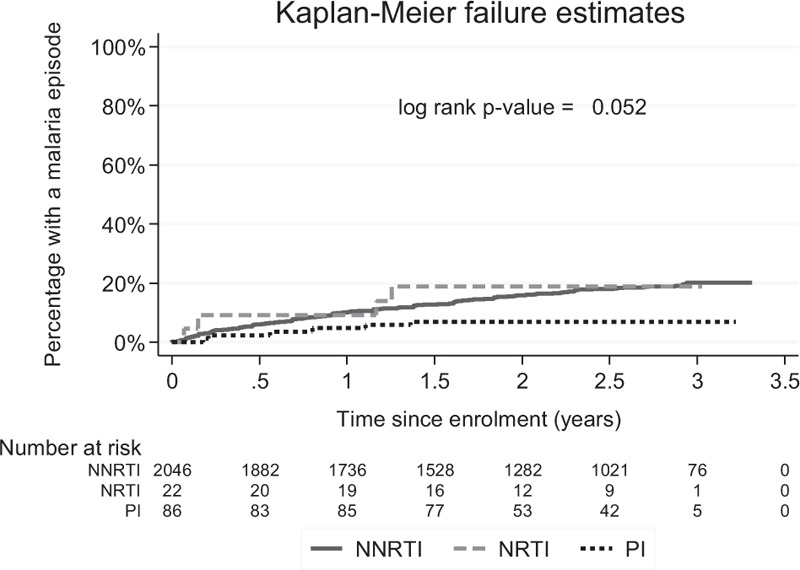
Time to occurrence of first malaria event by ART regimen.

ART, antiretroviral therapy; NNRTI, non-nucleoside reverse transcriptase inhibitor; NRTI, nucleoside reverse transcriptase inhibitor; PI, protease inhibitor.

Information on participants’ ART regimens at ART initiation and at enrolment was obtained from the records of the ART providers from where participants were recruited. During the trial, participants continued to receive ART from their usual providers, but trial staff ensured an uninterrupted supply of ART in case of unexpected shortages. Most participants were on an NNRTI-containing regimen (recommended first-line regimen in Uganda) [13], the remainder of participants were on regimens as outlined in Fig. 1.

Blood samples were used to prepare thick and thin films on a glass slide. Specimens were processed using Leishman's stain and examined by microscopy. Venous blood samples were taken for CD4^+^ cell counts and measured using a FACS-count system (Becton-Dickinson, San Jose, California USA).

### Analysis

Data were double entered and verified in MS Access and analysed using Stata, release 13 (StataCorp LP, College Station, Texas USA). Person years at risk were calculated from enrolment until the date last seen or end of trial. After each malaria episode, participants were considered to be not at risk for another episode until the episode resolved, or for 28 days, if a resolution date was not available. The time to the first malaria episode was examined using Kaplan–Meier methods and log rank tests to compare between groups. The incidence of malaria and rate ratios comparing ART regimens were calculated using random effects Poisson regression to account for multiple episodes within the same participant. Models were adjusted for treatment group (CTX or placebo), enrolment site, and time since enrolment as a priori confounders. ART regimens were categorized as NRTI (nucleoside reverse transcriptase) only, if a regimen containing only NRTIs was used; NNRTI containing, if one of the drugs was an NNRTI and none was a protease inhibitor; or protease inhibitor containing, if at least one drug was a protease inhibitor. ART regimen was analysed as a time-updated exposure. The effect of ART regimen on malaria incidence was examined overall and separately for both treatment groups; stratified rate ratios were obtained from a model containing a term for interaction between treatment group and ART regimen. Analyses were also stratified by current CD4^+^ cell count (<500 or ≥500) to assess whether the effect of ART regimen on malaria differed by CD4^+^ cell count.

## Results

Baseline characteristics have been described previously [11]. Briefly, the trial enrolled 2180 participants: 1002 in Entebbe and 1178 in Masaka. Half (1089) were randomised to CTX (stratified by site), 382 (18%) had CD4^+^ cell count less than 350 cells/μl at enrolment, and 569 (26%) were male. In total, 1721 (79%) participants had been on ART for at least 2 years. At the time of ART initiation, 2114 (97%) started on an NNRTI-containing regimen, 58 (3%) on an NRTI-only regimen, and three on a protease inhibitor -containing regimen; data were missing for five participants. At enrolment, 2046 (94%) were on an NNRTI containing regimen, 22 (1%) on an NRTI-only regimen and 86 (4%) on a protease inhibitor-containing regimen; enrolment ART information was missing for 26 participants (1%). Of those on a protease inhibitor-containing regimen, 75 (87%) were on lopinavir/ritonavir, 4 (5%) on atanazavir and 7 (8%) on another protease inhibitor. Of those on an NNRTI, 2066 (88%) were on nevirapine, 255 (12%) on efavirenz, and two on etravine. In total, 10 participants changed ART regimen during follow-up (nine NNRTI containing, one NRTI only); all changes were to a protease inhibitor-containing regimen (six to lopinavir/ritonavir, three to atanazavir and one to another protease inhibitor).

### Effect of antiretroviral therapy regimen

In total, 2154 participants contributed data to the analysis; 447 malaria episodes were observed during follow-up, of which 40 (8%) were diagnosed outside the study clinic. There was some evidence that time to the first malaria episode was shorter for participants on an NNRTI-containing or NRTI-only regimen compared with those on a protease inhibitor-containing regimen (*P* = 0.05; Fig. 1). In the unadjusted analysis, malaria incidence was similar for participants on NRTI-only compared with participants on a NNRTI-containing regimen, and was lower for participants on a protease inhibitor regimen compared with participants on an NNRTI (Table 1). After adjustment for treatment group, enrolment site and time since enrolment, malaria incidence among participants on an NRTI-only regimen was 1.6 (0.6–4.3) times higher than among those on an NNRTI-containing regimen, whereas that among participants on a protease inhibitor-containing regimen was 0.4 (0.2–1.0) times lower (*P* = 0.05; Table 1).

**Table 1:** Incidence of malaria by ART regimen overall and stratified by treatment arm and current CD4^+^ stratum.

	ART regimen^a^	Events	Person years	Rate^b^	Rate ratio^b^	Rate ratio^b,c^
					*P* = 0.02^d^	*P* = 0.05^b,d^
	NNRTI containing	435	4737	9.3 (8.3–10.4)	1	1
	NRTI only	5	50	9.9 (3.6–27.4)	1.1 (0.4–3.0)	1.6 (0.6–4.3)
	PI containing	7	202	3.5 (1.6–7.6)	0.4 (0.2–0.8)	0.4 (0.2–1.0)
Stratified by treatment arm					*P* = 0.92^e^	*P* = 0.95^e^
					*P* = 0.62^f^	*P* = 0.64^f^
CTX	NNRTI containing	97	2380	4.1 (3.3–5.0)	1	1
	NRTI only	1	25	4.2 (0.5–32.1)	1.0 (0.1–8.0)	1.5 (0.2–11.8)
	PI containing	2	100	2.0 (0.5–8.2)	0.5 (0.1–2.0)	0.5 (0.1–2.3)
					*P* = 0.07^f^	*P* = 0.11^f^
Placebo	NNRTI containing	338	2357	14.5 (12.9–16.4)	1	1
	NRTI only	4	25	15.4 (4.9–48.0)	1.1 (0.3–3.3)	1.6 (0.5–5.0)
	PI containing	5	102	5.0 (2.0–12.4)	0.3 (0.1–0.9)	0.4 (0.2–1.0)
Stratified by current CD4^+^ cell count					*P* = 0.86^e^	*P* = 0.96^e^
					*P* = 0.21^f^	*P* = 0.30^f^
CD4^+^ <500	NNRTI containing	200	2358	8.6 (7.5–10.1)	1	1
	NRTI only	2	33	7.0 (1.5–32.0)	0.8 (0.2–3.7)	1.4 (0.3–6.1)
	PI containing	4	118	3.5 (1.2–9.6)	0.4 (0.1–1.1)	0.5 (0.2–1.3)
					*P* = 0.21^f^	*P* = 0.24^f^
CD4^+^ ≥500	NNRTI containing	235	2379	9.9 (8.6–11.5)	1	1
	NRTI only	3	17	13.9 (3.6–54.4)	1.4 (0.4–5.5)	1.8 (0.5–6.6)
	PI containing	3	84	3.5 (1.1–11.6)	0.4 (0.1–1.2)	0.4 (0.1–1.4)

ART, antiretroviral therapy; CTX, cotrimoxazole; NNRTI, non-nucleoside reverse transcriptase; NRTI, nucleoside reverse transcriptase; PI, protease inhibitor.

^a^NNRTI containing was defined as a regimen that contained at least one NNRTI and no PI; NRTI only was defined as a regimen containing only NRTIs; PI containing was defined as a regimen in which at least one of the drugs was a PI.

^b^estimated from random effects Poisson regression.

^c^adjusted for treatment arm, site, and time since enrolment.

^d^*P* value for effect of ART regimen, from likelihood ratio test (LRT).

^e^LRT for interaction between treatment arm and ART regimen, and between treatment arm and CD4^+^ cell count group.

^f^*P* value from the Wald test.

In the adjusted analysis stratified by treatment group, malaria incidence among participants on an NRTI-only regimen was 1.5 (0.2–11.8) (CTX) and 1.6 (0.5–5.0) (placebo) times higher than those on an NNRTI-containing regimen, whereas that in participants on a protease inhibitor-containing regimen was 0.5 (0.1–2.3) (CTX) and 0.4 (0.2–1.0) (placebo) times lower, respectively. There was no evidence of interaction between the effect of treatment group and ART regimen (*P* = 0.95; Table 1). Rate ratios were similar in the analysis stratified by current CD4^+^ cell count, with no evidence of interaction between the effect of CD4^+^ cell count and ART regimen (*P* = 0.96; Table 1).

## Discussion

Protease inhibitor-containing regimens are recommended as second-line therapy for adults in Uganda and elsewhere [13,14] and were used by 4% of participants in this study. The most commonly used protease inhibitor was lopinavir/ritonavir. In total, 1% of participants were on an NRTI-only regimen, an alternative initial regimen recommended at the time [2,15].

We found that NRTI-only regimens provided the least protection against malaria followed by NNRTI-containing regimens. This is consistent with in-vitro studies that showed no antimalarial activity from NRTIs and some activity from NNRTIs but at levels which were not achievable *in vivo* at standard dosing [3,16]. Use of a protease inhibitor-containing regimen was associated with the strongest protection (rate ratio 0.4; 95% confidence interval = 0.2–1.0 *P* = 0.05, compared with NNRTI-containing regimens) and this is consistent with findings of a study in children [6].

We have previously reported that CTX use is associated with reduced incidence of malaria [17], an association that has been reported by other studies [18–20]. We did not find evidence of potentiation/interaction between the antimalarial effects of CTX and protease inhibitors.

In a study in children, the effect of protease inhibitors on malaria was partially attributed to a reduction in malaria recurrence as a result of increased lumefantrine levels after treatment, owing to cytochrome P450 enzyme inhibition by lopinavir/ritonavir [6]. These findings are in line with pharmacokinetic studies that have reported increased lumefantrine levels, in patients concomitantly taking lopinavir–ritonavir-based ART [21–24]. We did not measure drug levels during follow-up but we found that the time to first malaria episode (i.e. before malaria treatment) was longer for participants on a protease inhibitor-containing regimen implying that in adults, the observed effect might be because of a direct effect of protease inhibitors on *Plasmodium* proteases rather than drug–drug interactions with lumefantrine.

These findings are potentially of benefit. It has been suggested that the antimalarial prophylactic effect of protease inhibitor-containing ART regimens could reduce the cost of care in malaria endemic countries because of a potential reduction in malaria treatment costs [25], could help reduce the prevalence of malaria [26], could help reduce malaria transmission because of their gametocytocidal effect [27,28] and could even contribute to malaria eradication because lopinavir inhibits *Plasmodium falciparum* liver stage parasites [29], further clinical studies are, however, needed. Despite the lack of an antimalarial effect at therapeutic doses, benefits of NNRTI use, which include ease of dosing (because of combination pills), easier storage, better tolerability, and lower cost, should still make NNRTIs the preferred first-line choice in adults in malaria endemic areas [15,30]. However, clinicians may want to consider the added antimalarial benefit of using protease inhibitors when treating high-risk groups, for example, pregnant women, or when prescribing malaria prophylaxis in those on second-line therapy.

### Strength and limitations

Our study benefited from a well described study population that was followed regularly for up to 3.5 years and routinely assessed for malaria. However, although this offered an opportunity to investigate the effect of ART regimen on malaria, the COSTOP trial was not specifically designed to address this question. The number of participants on protease inhibitors was small, the observed protective effect just reached statistical significance, and therefore we cannot exclude the possibility that the effects might have occurred because of chance. As participants on protease inhibitors were on second-line therapy, their clinical condition may be different from those on first-line therapy; we were not able to adjust for potential confounders relating to individual health status. A history of fever (within previous 2 weeks) and parasitaemia were the basis for malaria diagnosis, this could have resulted in overestimation of cases, however 92% of cases were diagnosed by the study team, allowing for ascertainment of most diagnoses. Adherence to ART in our study population was high [11]. However, as we did not determine viral load or serum levels of ART drugs during follow-up, our findings may be subject to potential residual confounding resulting from differences in adherence between ART groups.

In conclusion, among HIV infected adults on ART, protease inhibitor-containing regimens were associated with reduced clinical malaria incidence compared with NNRTI-containing or NRTI-only regimens. The antimalarial properties of protease inhibitors may have clinical and public health importance.

## Acknowledgements

We are grateful to all the study participants and to the staff from the COSTOP study sites for their contribution, and to our ART providing partner institutions for their support in participants’ enrolment (The AIDS Support Organisation (TASO), Kisubi Hospital, Kitovu Mobile, Entebbe Hospital, Masaka Hospital, and Katabi Military Hospital). We acknowledge the valuable work of COSTOP Trial Monitors, the independent Trial Steering Committee, the independent Data Monitoring Committee, and the independent Endpoint Review Committee. Our work was supported through MRC (UK) grant number G0902150. This award was jointly funded by the UK Medical Research Council (MRC) and the UK Department for International Development (DFID) under the MRC/DFID Concordat agreement and is also part of the EDCTP2 programme supported by the European Union. KB receives support from the MRC UK and DFID (MRC grant number G0700837).

### Conflicts of interest

There are no conflicts of interest.
